# A novel method for controlling unobserved confounding using double confounders

**DOI:** 10.1186/s12874-020-01049-0

**Published:** 2020-07-22

**Authors:** Lu Liu, Lei Hou, Yuanyuan Yu, Xinhui Liu, Xiaoru Sun, Fan Yang, Qing Wang, Ming Jing, Yeping Xu, Hongkai Li, Fuzhong Xue

**Affiliations:** 1 Institute for Medical Dataology, Shandong University, 250012 Jinan, Shandong People’s Republic of China; 2 Department of Biostatistics, School of Public Health, Cheeloo College of Medicine, Shandong University, 250012 Jinan, Shandong People’s Republic of China; 3 Synthesis Electronic Technology Co.Ltd, 250012 Jinan, Shandong People’s Republic of China

**Keywords:** Unobserved confounders, Generalized moment estimate model, Identification, Causal effect

## Abstract

**Background:**

Controlling unobserved confounding still remains a great challenge in observational studies, and a series of strict assumptions of the existing methods usually may be violated in practice. Therefore, it is urgent to put forward a novel method.

**Methods:**

We are interested in the causal effect of an exposure on the outcome, which is always confounded by unobserved confounding. We show that, the causal effect of an exposure on a continuous or categorical outcome is nonparametrically identified through only two independent or correlated available confounders satisfying a non-linear condition on the exposure. Asymptotic theory and variance estimators are developed for each case. We also discuss an extension for more than two binary confounders.

**Results:**

The simulations show better estimation performance by our approach in contrast to the traditional regression approach adjusting for observed confounders. A real application is separately applied to assess the effects of Body Mass Index (BMI) on Systolic Blood Pressure (SBP), Diastolic Blood Pressure (DBP), Fasting Blood Glucose (FBG), Triglyceride (TG), Total Cholesterol (TC), High Density Lipoprotein (HDL) and Low Density Lipoprotein (LDL) with individuals in Shandong Province, China. Our results suggest that SBP increased 1.60 (95% CI: 0.99–2.93) mmol/L with per 1- kg/m^2^ higher BMI and DBP increased 0.37 (95% CI: 0.03–0.76) mmol/L with per 1- kg/m^2^ higher BMI. Moreover, 1- kg/m^2^ increase in BMI was causally associated with a 1.61 (95% CI: 0.96–2.97) mmol/L increase in TC, a 1.66 (95% CI: 0.91–55.30) mmol/L increase in TG and a 2.01 (95% CI: 1.09–4.31) mmol/L increase in LDL. However, BMI was not causally associated with HDL with effect value − 0.20 (95% CI: − 1.71–1.44). And, the effect value of FBG per 1- kg/m^2^ higher BMI was 0.56 (95% CI: − 0.24–2.18).

**Conclusions:**

We propose a novel method to control unobserved confounders through double binary confounders satisfying a non-linear condition on the exposure which is easy to access.

## Background

Controlling unobserved confounding is a great challenge when estimating the causal effect of an exposure on an outcome of interest in observational studies [1–4]. Several techniques such as traditional regression model, marginal structure model, adjustment, stratification, inverse probability weighing (IPW), matching based on propensity score cannot deal with unobserved confounding [5–9]. The observed data distribution may have compatibility with many contradictory causal explanations due to the existence of unobserved confounding. In this circumstance, we say the causal estimand is not identified. On the contrary, when the causal estimand can be obtained entirely from observable probability distributions, we say the query is identified.

Some methods have been developed to alleviate the problems caused by unobserved confounding. Instrumental variable analysis (IVA) is the commonly used method to eliminate unobserved confounding [10]. But in practice, choosing valid instruments (IVs) is a stumbling block in IVA [11–13]. The difference-in-differences (DID) is contingent on the availability of repeated outcomes in both periods, but invokes strict parallel trend assumptions, i.e., confounders varying across the groups are time invariant and time-varying confounders are group invariant [14, 15]. Regression discontinuity design (RDD) is a quasi-experimental pretest-posttest design for controlling unobserved confounding by assigning a cutoff or threshold above or below to a treatment [16]. Nevertheless, RDD requires that treatment assignment is sufficiently randomized at the threshold [17]. Negative controls are widely used in epidemiologic practice to detect the presence of unobserved confounding. While a valid negative control outcome needs to be influenced by the same unobserved confounders of the exposure effects on the outcome in view, although not directly influenced by the exposure. But this approach fails to obtain causal estimation of the exposure on the outcome [18]. Moreover, strict assumptions of the methods above usually may be violated in practice and impose restrictions on their generalization.

In this article, we propose a novel method to control unobserved confounding through double confounders with two values satisfying a non-linear condition on the exposure. Under the assumption of ignorable treatment assignment, causal effects can be identified and estimated using commonly generalized moment estimate model. Furthermore, we relax the assumption that observed and unobserved confounders are independent in sensitivity analysis and observe that even when the correlations between binary observed confounders and unobserved confounders are relatively weak, we still obtain the almost unbiased causal effect estimation. Additionally, we explore the statistical properties of this method by a simulation study and compare with the traditional regression approach only adjusting for observed confounders. Finally, we apply this method to a cohort from a follow-up survey (136,895 individuals) from 2007 to 2015 in Jining, China to exam the causal associations of BMI on other factors, including SBP, DBP, FBG, TG, TC, HDL and LDL.

## Methods

### Notation and preliminaries

**Fig. 1:**
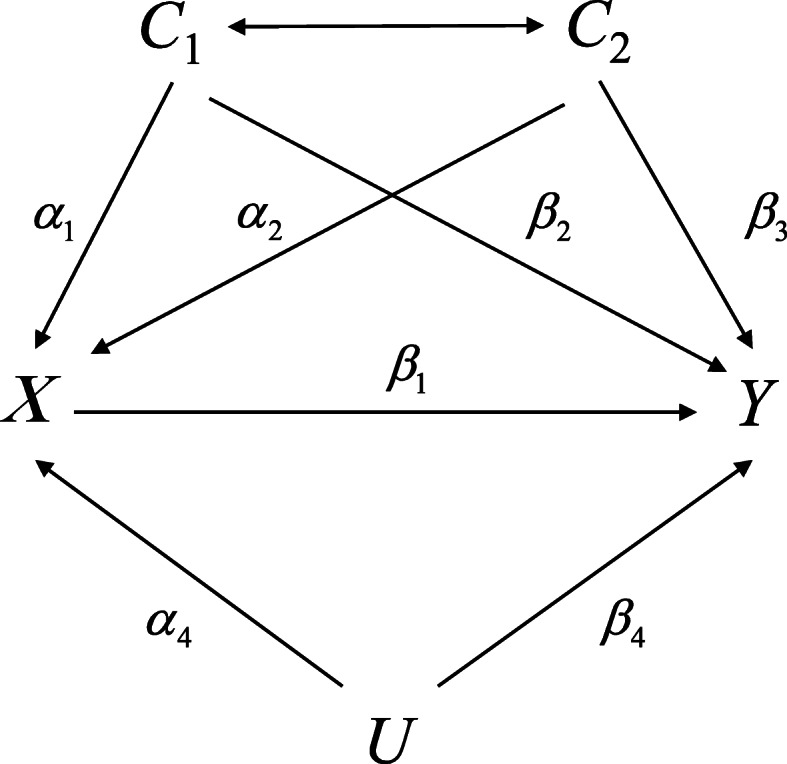
Causal diagram for *X* (treatment) and *Y* (outcome) with *C*_1_, *C*_2_ (observed confounders) and *U* (other unobserved confounders)

Throughout, we let *X*, *Y*, *C*_1_, *C*_2_ and *U* denote the treatment, outcome, two observed confounders and unobserved confounders, respectively (Fig. 1). Following the convention in causal inference, we use *Y*(*x*) to denote the potential outcome of *Y* under an intervention which sets *X* to *x*, and the observed outcome *Y* is a realization of the potential outcome under the exposure actually received: *Y* = *Y*(*x*) when *X* = *x*. We focus on the average causal effect (ACE) of *X* on *Y* which is the difference in expectation of potential outcome at two different exposure levels 0 and 1, for instance, *ACE*_*X* → *Y*_ = *E*(*Y*(1) − *Y*(0)) for a binary exposure. The conditional ignorability assumption *Y*(*x*) ⊥ *x* ∣ *C*_1_, *C*_2_ is conventionally made in causal inference, but it does not hold in the present of unmeasured confounding *U*. In this case, latent ignorability *Y*(*x*) ⊥ *x* ∣ *C*_1_, *C*_2_, *U* is more reasonable, allowing for an unobserved confounder *U* that captures the source of non-ignorability of the exposure mechanism. The model with continuous exposure and outcome can be developed as follows.

1 $$ X=F\left({C}_1,{C}_2\right)+\varphi \left(U,{\varepsilon}_X\right) $$

2 $$ Y={\beta}_0+{\beta}_1X+{\beta}_2{C}_1+{\beta}_3{C}_2+\phi \left(U,{\varepsilon}_Y\right) $$

where *ε*_*Y*_ and *ε*_*X*_ are two mutually independent random errors with means 0 and variances ${\sigma}_Y^2$ and ${\sigma}_X^2$ respectively, *φ*(·) and *ϕ*(·) are two arbitrary functions. The causal effect of *X* on *Y* is interpreted as *β*_1_. Similarly, for binary exposure and outcome, we can also construct corresponding linear probability model (LPM) for *P*(*X* = 1| *C*_1_, *C*_2_, *U*), *P*(*Y* = 1| *C*_1_, *C*_2_, *U*), respectively. In addition, the “complementary log link” of transformation of *Y* (i.e. − log(1 − *Risk*), *Risk* denotes cumulative completion risk) is also appropriate for our method [19].

### Identification and estimation of causal effects with double binary confounders

Obviously, the estimation of regression model adjusting for *C*_1_ and *C*_2_ is biased when there exists an unobserved confounder *U*. Unfortunately, the no unobserved confounding assumption usually not be satisfied in practical studies. Next, we explain how to identify and estimate the causal effect by relaxing the no unobserved confounding assumption in four cases with different types of exposure and outcome (continuous or binary). It will be shown below that causal effect *β*_1_ in (2) is not identifiable if the function *F*(*X*| *C*_1_, *C*_2_) is linear with respect to *C*_1_ and *C*_2_.

Within the causal framework provided by Fig. 1, we propose four assumptions and discuss the necessary and sufficient condition for identification of parameters in the model (2).

**Assumption 1**: *E*(*ϕ*(*U*, *ε*_*Y*_)) = 0.

**Assumption 2**: (*C*_1_, *C*_2_) ⊥ *ϕ*(*U*, *ε*_*Y*_), i.e. (*C*_1_, *C*_2_) are not associated with any confounder (*U*) of the exposure–outcome relationship and random errors *ε*_*Y*_.

**Assumption 3:** The effect of (X, *C*_1_, *C*_2_) on *Y* is linear (or no interaction).

**Assumption 4:** The effect of (*C*_1_, *C*_2_) on *X* is non-linear (e.g. with an interaction effect or the quadratic term of *C*_1_ and *C*_2_ on *X*).

Under Assumptions 1 and 2, *E*(*ϕ*(*U*, *ε*_*Y*_)| *C*_1_, *C*_2_) = 0 is satisfied. The **Assumption 2** is the same as the exchangeability assumption in instrumental variable (IV) analysis – an IV is not associated with any confounder of the exposure–outcome relationship. In addition, a valid IV must have a main effect on *X* and no direct effect on *Y*, which are called the Relevance and the Exclusion restriction assumption, respectively [20]. In our method, (*C*_1_, *C*_2_) can be regarded as two near-IVs with a non-linear effect on *X* and a linear effect on *Y* as stated in the **Assumption 3** and **4**.

**Theorem 1**: *The causal effect β*_1_*of X on Y in model (**2**) is identifiable if and only if the Assumptions 1–4 are satisfied.*

See Appendix A for the proof of Theorem 1.

Causal effect is identified based on above four assumptions. And various estimation approaches have been applied to estimate the causal effect, including moment estimation, maximum likelihood estimation and generalized moment estimate model (GMM) with different assumptions [21–23]. In this section, we aim to find an efficient estimation of causal effect in the model (2).

In our estimation approach, the pivotal “orthogonality condition” is the independence (*C*_1_, *C*_2_) ⊥ *ϕ*(*U*, *ε*_*Y*_), which implies the following equation:

3 $$ E\left[\left(Y-{\beta}_0-{\beta}_1X-{\beta}_2{C}_1-{\beta}_3{C}_2\right)f\left({C}_1,{C}_2\right)\right]=\mathbf{0}, $$

where *f*(·) = (*f*_1_(·), ⋯, *f*_*K*_(·))^*T*^ is an arbitrary vector function and **0** is a *K* × 1 zero vector.

For the case of confounders *C*_1_ and *C*_2_ with two values, we chose the function *f*^∗^(*C*_1_, *C*_2_) = (*δ*(*C*_1_ = 0, *C*_2_ = 0), *δ*(*C*_1_ = 0, *C*_2_ = 1), *δ*(*C*_1_ = 1, *C*_2_ = 0), *δ*(*C*_1_ = 1, *C*_2_ = 1))^*T*^, we have

4 $$ E\left[\left(Y-{\beta}_0-{\beta}_1X-{\beta}_2{C}_1-{\beta}_3{C}_2\right){f}^{\ast}\left({C}_1,{C}_2\right)\right]=\mathbf{0},\kern0.5em $$

where **0** is a 4 × 1 zero vector. Define ***β*** = (*β*_0_, *β*_1_, *β*_2_, *β*_3_)^*T*^,

$$ {R}^{\ast }=\left(\begin{array}{l}E\left( Y\delta \left({C}_1=0,{C}_2=0\right)\right)\\ {}E\left( Y\delta \left({C}_1=0,{C}_2=1\right)\right)\\ {}E\left( Y\delta \left({C}_1=1,{C}_2=0\right)\right)\\ {}E\left( Y\delta \left({C}_1=1,{C}_2=1\right)\right)\end{array}\right),\kern0.5em {Q}^{\ast }=\left(\begin{array}{l}E\left(\delta \left({C}_1=0,{C}_2=0\right)\right)\kern0.5em E\left( X\delta \left({C}_1=0,{C}_2=0\right)\right)\kern0.5em E\left({C}_1\delta \left({C}_1=0,{C}_2=0\right)\right)\kern0.5em E\left({C}_2\delta \left({C}_1=0,{C}_2=0\right)\right)\ \\ {}E\left(\delta \left({C}_1=0,{C}_2=0\right)\right)\kern0.5em E\left( X\delta \left({C}_1=0,{C}_2=1\right)\right)\kern0.5em E\left({C}_1\delta \left({C}_1=0,{C}_2=1\right)\right)\kern0.75em E\left({C}_2\delta \left({C}_1=0,{C}_2=1\right)\right)\kern0.5em \\ {}E\left(\delta \left({C}_1=0,{C}_2=0\right)\right)\kern0.5em E\left( X\delta \left({C}_1=1,{C}_2=0\right)\right)\kern0.5em E\left({C}_1\delta \left({C}_1=1,{C}_2=0\right)\right)\kern0.75em E\left({C}_2\delta \left({C}_1=1,{C}_2=0\right)\right)\ \\ {}E\left(\delta \left({C}_1=0,{C}_2=0\right)\right)\kern0.5em E\left( X\delta \left({C}_1=1,{C}_2=1\right)\right)\kern0.75em E\left({C}_1\delta \left({C}_1=1,{C}_2=1\right)\right)\kern0.9000001em E\left({C}_2\delta \left({C}_1=1,{C}_2=1\right)\right)\kern0.5em \end{array}\right), $$

the Eq. (4) can be rewritten as *R*^∗^ − *Q*^∗^***β*** = 0. Solve for GMM estimator

$$ \hat{{\boldsymbol{\beta}}^{\ast }}={\hat{Q^{\ast}}}^{-1}\hat{R^{\ast }}, $$

where the elements of $\hat{Q^{\ast }}$ and $\hat{R^{\ast }}$ are sample means about *X*, *C*_1_, *C*_2_ and *C*_1_, *C*_2_, *Y*, respectively. Thus $\hat{{\boldsymbol{\beta}}^{\ast }}$ is a valid estimator only if *Q*^∗^ has full rank. The determinant of the matrix *Q*^∗^ is

$$ {\displaystyle \begin{array}{l}\det \left({Q}^{\ast}\right)=P\left({C}_1=0,{C}_2=0\right)P\left({C}_1=0,{C}_2=1\right)P\left({C}_1=1,{C}_2=0\right)P\left({C}_1=1,{C}_2=1\right)\\ {}\cdot \left\{\left[E\left(X|{C}_1=0,{C}_2=1\right)-E\left(X|{C}_1=0,{C}_2=0\right)\right]-\left[E\left(X|{C}_1=1,{C}_2=1\right)-E\left(X|{C}_1=1,{C}_2=0\right)\right]\right\}.\end{array}} $$

Note that the nonlinearity of *E*[*X*| *C*_1_, *C*_2_] with respect to *C*_1_ and *C*_2_ implies

$$ \kern0.1em \left[E\left(X|{C}_1=0,{C}_2=1\right)-E\left(X|{C}_1=0,{C}_2=0\right)\right]-\left[E\left(X|{C}_1=1,{C}_2=1\right)-E\left(X|{C}_1=1,{C}_2=0\right)\right]\ne 0. $$

Furthermore, the estimated causal effect $\hat{\beta_1}$ is

$$ \hat{\beta_1}=\frac{\left[E\left(Y|{C}_1=1,{C}_2=1\right)-E\left(Y|{C}_1=1,{C}_2=0\right)\right]-\left[E\left(Y|{C}_1=0,{C}_2=1\right)-E\left(Y|{C}_1=0,{C}_2=0\right)\right]}{\left[E\left(X|{C}_1=0,{C}_2=1\right)-E\left(X|{C}_1=0,{C}_2=0\right)\right]-\left[E\left(X|{C}_1=1,{C}_2=1\right)-E\left(X|{C}_1=1,{C}_2=0\right)\right]}. $$

The estimated causal effect $\hat{\beta_1}$ is reasonable compared with the “ratio estimator” of a binary IV when *C*_1_ and *C*_2_ are regarded as two near-IVs. Denote the sub-sample average of *y* and *x* by ${\overline{y}}_1$ and ${\overline{x}}_1$ when *z* = 1 and by ${\overline{y}}_0$ and ${\overline{x}}_0$ when *z* = 0. Then $\varDelta y/\varDelta z={\overline{y}}_1-{\overline{y}}_0$ and $\varDelta x/\varDelta z={\overline{x}}_1-{\overline{x}}_0$, and “ratio estimator” of an IV [24] is

$$ {\hat{\beta}}_{IV}=\frac{{\overline{y}}_1-{\overline{y}}_0}{{\overline{x}}_1-{\overline{x}}_0}. $$

Define *g*(***β***) = *E*[(*Y* − *β*_0_ − *β*_1_*X* − *β*_2_*C*_1_ − *β*_3_*C*_2_)*f*(*C*_1_, *C*_2_)], and ***β*** can be identified only when *rank*(*Q*) = *l* + 2, *l* = 2, where *l* denotes the number of the observed confounders. For any *f*(·) that makes *rank*(*Q*) = *l* + 2, a GMM estimate of ***β*** is

5 $$ \hat{\boldsymbol{\beta}}=\arg\ \min \left\{\hat{g}\left(\boldsymbol{\beta} \right)\hbox{'}\hat{W}\hat{g}\left(\boldsymbol{\beta} \right)\right\}={\left(\hat{Q}\hbox{'}\hat{W}\hat{Q}\right)}^{-1}\hat{Q}\hbox{'}\hat{W}\hat{R}, $$

where $\hat{W}$ is denoted as a symmetric and positive definite weight matrix for *K* ≥ (*l* + 2), *K* = rank(*Q*). The elements of $\hat{g}\left(\boldsymbol{\beta} \right),\hat{Q}$ and $\hat{R}$ are sample means of the corresponding elements of *g*(***β***), *Q* and *R* respectively [20].

If $\hat{W}\to W$ as *N* → ∞ in probability where *W* is positive semi-definite and *N* denotes the sample size. The main general properties about GMM estimators under the appropriate regularity conditions are that,

1. $\hat{\boldsymbol{\beta}}\to \boldsymbol{\beta}$ in probability as *N* → ∞ where ***β*** denotes the true parameter.

2. $\sqrt{N}\left(\hat{\boldsymbol{\beta}}-\boldsymbol{\beta} \right)$ converges in distribution to a normal distribution with mean zero and variance ${\left({Q}^T WQ\right)}^{-1}{Q}^T WE\left[f\left({C}_1,{C}_2\right)f{\left({C}_1,{C}_2\right)}^T\right] WQ{\left({Q}^T WQ\right)}^{-1}{\sigma}_{res}^2$, where ${\sigma}_{res}^2$ is the variance of *ϕ*(*U*, *ε*_*Y*_).

Then we describe a GMM estimator with the efficient instrument (a function of *C*_1_, *C*_2_) proposed by Newey and McFadden [25], which has the minimum variance among all estimators satisfying the Eq. (4). It also shown that there is an efficient estimator $\hat{\boldsymbol{\beta}}$ when the instrument *f*(*C*_1_, *C*_2_) is defined as

$$ {f}^{\mathrm{eff}}\left({C}_1,{C}_2\right)={\left\{E\left[\frac{\partial \psi \left(X,{C}_1,{C}_2,Y\right)}{\partial \boldsymbol{\beta}}\right]|{C}_1,{C}_2\right\}}^T={\left(1,E\left[X|{C}_1,{C}_2\right],{C}_1,{C}_2\right)}^T. $$

From (5), the efficient GMM estimator of ***β*** is defined as ${\hat{\boldsymbol{\beta}}}^{\mathrm{eff}}={\left({\left({\hat{Q}}^{\mathrm{eff}}\right)}^T\hat{W}{\hat{Q}}^{\mathrm{eff}}\right)}^{-1}{\left({\hat{Q}}^{\mathrm{eff}}\right)}^T\hat{W}{\hat{R}}^{\mathrm{eff}}$, where ${\hat{Q}}^{\mathrm{eff}}$ and ${\hat{R}}^{\mathrm{eff}}$ are the sample means of

$$ {\hat{Q}}^{\mathrm{eff}}=\left[\begin{array}{l}\kern1em 1\kern4em E(X)\kern3.75em E\left({C}_1\right)\kern1.5em E\left({C}_2\right)\\ {}E(X)\kern0.75em E\left[E{\left(X|{C}_1,{C}_2\right)}^2\right]\kern0.75em E\left({C}_1X\right)\kern0.75em E\left({C}_2X\right)\\ {}E\left({C}_1\right)\kern2.5em E\left({C}_1X\right)\kern3em E\left({C}_1^2\right)\kern1.5em E\left({C}_1{C}_2\right)\\ {}E\left({C}_2\right)\kern2.5em E\left({C}_2X\right)\kern2.75em E\left({C}_1{C}_2\right)\kern0.75em E\left({C}_2^2\right)\end{array}\right]\kern0.5em {\hat{R}}^{\mathrm{eff}}=\left[\begin{array}{l}\kern2.75em E(Y)\\ {}E\left[ YE\left(X|{C}_1,{C}_2\right)\right]\\ {}\kern2.25em E\left({C}_1Y\right)\\ {}\kern2.25em E\left({C}_2Y\right)\end{array}\right]. $$

The matrix *Q*^eff^ equals *E*[*f*(*C*_1_, *C*_2_)*f*(*C*_1_, *C*_2_)^*T*^] proved in Appendix B and has full rank if the nonlinearity condition in Theorem 1 holds. Since *Q*^eff^ and the positive definite $\hat{W}$ for *l* = 2 are full rank, ${\hat{\boldsymbol{\beta}}}^{\mathrm{eff}}$ is a valid estimator which can be simplified as

6 $$ {\hat{\boldsymbol{\beta}}}^{\mathrm{eff}}={\left({\hat{Q}}^{\mathrm{eff}}\right)}^{-1}{\hat{W}}^{-1}{\left[{\left({\hat{Q}}^{\mathrm{eff}}\right)}^T\right]}^{-1}{\left({\hat{Q}}^{\mathrm{eff}}\right)}^T\hat{W}{\hat{R}}^{\mathrm{eff}}={\left({\hat{Q}}^{\mathrm{eff}}\right)}^{-1}{\hat{R}}^{\mathrm{eff}}. $$

We find ${\hat{\boldsymbol{\beta}}}^{\mathrm{eff}}$ does not rely on the choice of $\hat{W}$ at this point. From the above property of the GMM estimator, the asymptotic variance of ${\hat{\boldsymbol{\beta}}}^{\mathrm{eff}}$ is easily obtained by *Q*^eff^ and ${\sigma}_{res}^2$

$$ AVAR\left({\hat{\boldsymbol{\beta}}}^{\mathrm{eff}}\right)={\left({\hat{Q}}^{\mathrm{eff}}\right)}^{-1}E\left[{f}^{\mathrm{eff}}\left({C}_1,{C}_2\right){f}^{\mathrm{eff}}{\left({C}_1,{C}_2\right)}^T\right]{\left({\hat{Q}}^{\mathrm{eff}}\right)}^{-1}{\sigma}_Y^2={\left({\hat{Q}}^{\mathrm{eff}}\right)}^{-1}{\sigma}_{res}^2. $$

Additionally, we show for two binary observed confounders that any *f*(·) which makes the Eq. (4) have the unique solution leads to the same estimator of parameters as that obtained by *f*^∗^(*C*_1_, *C*_2_) in Appendix B. Therefore, for the case of two binary observed confounders, our estimator $\hat{{\boldsymbol{\beta}}^{\ast }}$ is efficient and it is not necessary to choose an extra function *f*(·) to improve the efficiency.

### Extend to the case of more than two binary confounders

Note that the method proposed is an easy tool to detect the causal effect in the absence of enough confounding information. Because of a growing appreciation of the power gains of multivariate association analyses, more than two covariates are generally selected to analysis in practice. In this section, for the case of more than two binary confounders, we discuss the identification of parameter *β*_1_ in the following model

7 $$ Y={\beta}_0+{\beta}_1X+{\beta}_2{C}_1+{\beta}_3{C}_2+\cdots +{\beta}_{l+1}{C}_l+\phi \left(U,{\varepsilon}_Y\right). $$

Define ***β*** = (*β*_0_, *β*_1_, *β*_2_, ⋯, *β*_*l* + 1_)^*T*^, Eq. (7) can be rewritten as *Q* × ***β*** = *R*, where *Q* = (1, *E*(*X*| *C*_1_, *C*_2_, ⋯*C*_*l*_), *C*_1_, *C*_2_, ⋯*C*_*l*_) denotes a 2^*l*^ × (*l* + 2) matrix, ***β*** is a (*l* + 2) vector. According to the equation *Q* × ***β*** = *R*, parameter *β*_1_ is identifiable if *K* = rank(*Q*) ≥ (*l* + 2), such that the matrix *Q* has full column rank. We extend **Assumptions 2–4** to the case of more than two binary confounders as follows:

Assumption 2*: (*C*_1_, *C*_2_, ⋯, *C*_*l*_) ⊥ *ϕ*(*U*, *ε*_*Y*_), i.e. (*C*_1_, *C*_2_, ⋯, *C*_*l*_) are not associated with any confounder (*U*) of the exposure–outcome relationship and random errors *ε*_*Y*_.

Assumption 3*: The effect of (*X*, *C*_1_, *C*_2_, ⋯, *C*_*l*_) on *Y* is linear.

Assumption 4*: The effect of (*C*_1_, *C*_2_, ⋯, *C*_*l*_) on *X* is non-linear (e.g. with an interaction effect or the quadratic term of (*C*_1_, *C*_2_, ⋯, *C*_*l*_) on *X*).

Thus, under the **Assumption 1** and the Assumptions 2*-4*, the causal effect of *X* on *Y* can be identified.

Similar to the case of two binary observed confounders *C*_1_ and *C*_2_, different *f*(·) for *C*_1_, *C*_2_, ⋯, *C*_*l*_ in (7) leads to different estimators. Here, we have

8 $$ E\left[\left(Y-{\beta}_0-{\beta}_1X-{\beta}_2{C}_1-{\beta}_3{C}_2-\cdots -{\beta}_{l+1}{C}_l\right)f\left({C}_1,{C}_2,\cdots, {C}_l\right)\right]=\mathbf{0}. $$

Under the conditional expectation (8), the efficient GMM estimator ${\hat{\boldsymbol{\beta}}}^{\mathrm{eff}}$ of ***β*** is ${\hat{\boldsymbol{\beta}}}^{\mathrm{eff}}={\left({\hat{Q}}^{\mathrm{eff}}\right)}^{-1}{\hat{R}}^{\mathrm{eff}}$, with

$$ {f}^{\mathrm{eff}}\left({C}_1,\cdots {C}_l\right)={\left\{E\left[\frac{\partial \psi \left(X,{C}_1,\cdots {C}_l,Y\right)}{\partial \boldsymbol{\beta}}\right]|{C}_1,\cdots {C}_l\right\}}^T={\left(1,E\left[X|{C}_1,\cdots {C}_l\right],{C}_1,\cdots {C}_l\right)}^T. $$

From the above property of the GMM estimator, we can obtain the asymptotic variance of ${\hat{\boldsymbol{\beta}}}^{\mathrm{eff}}$ in this case

$$ AVAR\left({\hat{\boldsymbol{\beta}}}^{\mathrm{eff}}\right)={\left({\hat{Q}}^{\mathrm{eff}}\right)}^{-1}E\left[{f}^{\mathrm{eff}}\left({C}_1,\cdots, {C}_l\right){f}^{\mathrm{eff}}{\left({C}_1,\cdots, {C}_l\right)}^T\right]{\left({\hat{Q}}^{\mathrm{eff}}\right)}^{-1}{\sigma}_Y^2={\left({\hat{Q}}^{\mathrm{eff}}\right)}^{-1}{\sigma}_{res}^2, $$

where ${\hat{Q}}^{\mathrm{eff}}$ and ${\hat{R}}^{\mathrm{eff}}$ are the sample means of

$$ {Q}^{eff}=\left[\begin{array}{l}\kern1em 1\kern5.25em E(X)\kern4em E\left({C}_1\right)\kern1em \cdots \kern0.75em E\left({C}_l\right)\\ {}E(X)\kern0.75em E\left[E{\left(X|{C}_1,\cdots, {C}_l\right)}^2\right]\kern0.75em E\left({C}_1X\right)\kern0.5em \cdots \kern0.5em E\left({C}_lX\right)\\ {}E\left({C}_1\right)\kern3.5em E\left({C}_1X\right)\kern3.5em E\left({C}_1^2\right)\kern0.75em \cdots \kern0.5em E\left({C}_1{C}_l\right)\\ {}\kern1em \vdots \kern6.25em \vdots \kern6.25em \vdots \kern5.25em \vdots \\ {}E\left({C}_l\right)\kern3.5em E\left({C}_lX\right)\kern3.25em E\left({C}_1{C}_l\right)\kern0.5em \cdots \kern0.75em E\left({C}_l^2\right)\end{array}\right],\kern0.5em {R}^{eff}=\left[\begin{array}{l}\kern2.75em E(Y)\\ {}E\left[ YE\left(X|{C}_1,\cdots, {C}_l\right)\right]\\ {}\kern2.25em E\left({C}_1Y\right)\\ {}\kern3.75em \vdots \\ {}\kern2.25em E\left({C}_lY\right)\end{array}\right]. $$

### Simulations

In order to investigate the performance of our method in simulation study, as well as to determine in which scenario it performs well or badly in comparison with the traditional regression approach adjusting for observed confounders, we perform simulations with four cases. In our simulations, data are generated based on the causal diagram depicted in Fig. 1.

In each case (exposure and outcome are continuous or binary respectively, denoted as Simulation A: exposure and outcome are both continuous; Simulation B: exposure is binary while outcome is continuous; Simulation C: exposure is continuous while outcome is binary; Simulation D: exposure and outcome are both binary), we take into account two scenarios where *C*_1_, *C*_2_ and *U* are independent, *C*_1_, *C*_2_ and *U* are correlated respectively (For Simulation A, two scenarios denotes as Simulation A1, Simulation A2, respectively, similar notation for Simulation B, C, D). We performed simulation to compare performances of three methods including crude association without adjusting for *C*_1_, *C*_2_ and *U* (model 1), model of adjusting for *C*_1_ and *C*_2_ (model 2) and the method we proposed here (model 3). We simulated baseline covariates and a quantitative exposure in a large population consisting of 2000 subjects in Simulation A, 10000 subjects in Simulation B and 100,000 subjects in Simulation C, D.

The data of Simulation A1 were generated for each individual in the following procedure:

1. *C*_1_~*Bernoulli*(*N*, 0.5), *C*_2_~*Bernoulli*(*N*, 0.5).
2. *U*~*N*(0, 1).
3. *ε*_*X*_~*U*(0, 1), *ε*_*Y*_~*U*(0, 1).
4. *X* = 0.6*C*_1_ + 0.4*C*_2_ + 0.6*C*_1_*C*_2_ + 0.2*U* + *ε*_*X*_.
5. *Y* = *X* + 0.4*C*_1_ + 0.3*C*_2_ + 0.1*U* + *ε*_*Y*_.

The data of Simulation A2 were generated for each individual in the following procedure:

1. $$ \left({C}_1,{C}_2,U\right)\sim N\left(\mu, \Sigma \right),\mu =\left(\begin{array}{l}0\\ {}0\\ {}0\end{array}\right),\Sigma =\left[\begin{array}{l}1\kern1em \mathrm{d}\kern1em \mathrm{c}\\ {}\mathrm{d}\kern1em 1\kern1em \mathrm{c}\\ {}\mathrm{c}\kern1em \mathrm{c}\kern1em 1\end{array}\right],c=0.1,d=0.2 $$.
2. Draw *C*_1_ from the first column of multivariate normal distribution, and discretize it into a binary variable with probability*P*(*C*_1_ = 1) = 0.5.
3. Draw *C*_2_ from the second column of multivariate normal distribution, and discretize it into a binary variable with probability*P*(*C*_2_ = 1) = 0.5.
4. Draw *U* from the third column of multivariate normal distribution.
5. *ε*_*X*_~*U*(0, 1), *ε*_*Y*_~*U*(0, 1).
6. *X* = 0.6*C*_1_ + 0.4*C*_2_ + 0.6*C*_1_*C*_2_ + 0.2*U* + *ε*_*X*_.
7. *Y* = *X* + 0.4*C*_1_ + 0.3*C*_2_ + 0.1*U* + *ε*_*Y*_.

The data of Simulation B1-B*2*, Simulation C1-C2, Simulation D1-D2 were similarly generated for each individual. Details are listed in the Appendix C.

### Data application

Numerous epidemiological studies have evaluated the relationships between BMI and SBP, DBP, FBG, TG, TC, HDL and LDL, respectively, but the causal effects are inconclusive due to the existence of unobserved confounding [26–28]. Hence, we use the method proposed in this article to evaluate the potential causal effect of BMI on SBP, DBP, FBG, TG, TC, HDL and LDL compared with the traditional regression approach adjusting for age (discrete) and gender, adjusting for age (continuous) and gender and adjusting for age (discrete), gender and other health factors (including SBP, DBP, FBG, TC, TG, HDL and LDL) using data from a follow-up survey in Jining, Shandong Province.

## Results

### Simulation results

We separately varied across the main effect value *α*_1_ of *C*_1_ on *X*, the main effect value *α*_2_ of *C*_2_ on *X*, the interaction effect *α*_3_ of *C*_1_ and *C*_2_ on *X*, the causal effect *β*_1_ of *X* on *Y*, the confounding effect *β*_2_ of *C*_1_ on *Y*, the correlation coefficient *c* between *C*_1_ and *C*_2_ as well as correlation coefficient *d* between *C*_1_ and *U*.

**Fig. 2:**
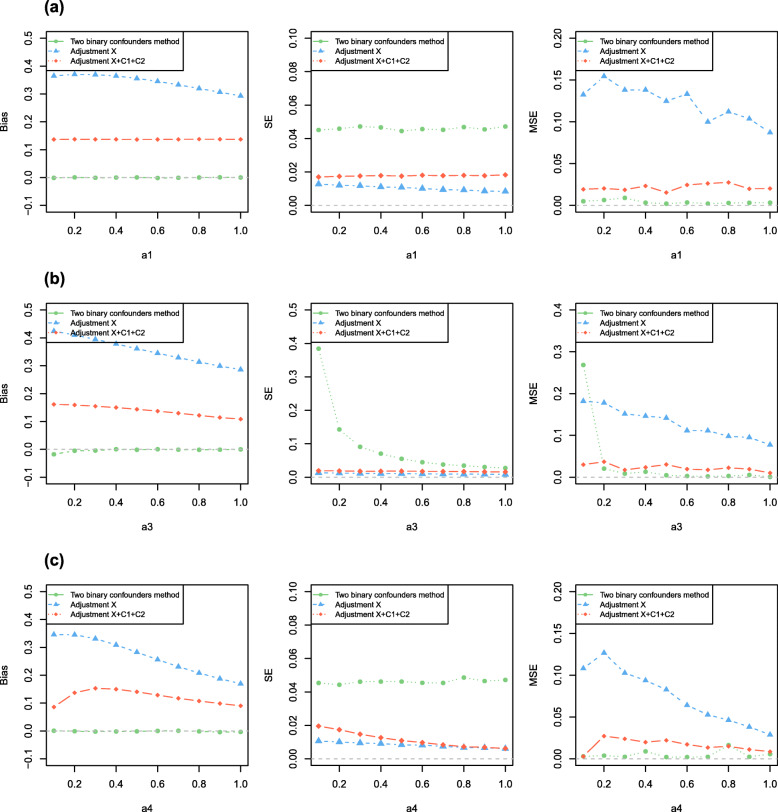
The Simulation A1 result. Results shows the estimated biases, *SE* and *MSE* from the 3 models for varied effects of **a***C*_1_ on *X*, **b** the interaction effect of *C*_1_ and *C*_2_ on *X*, **c***U* on *X*

**Fig. 3:**
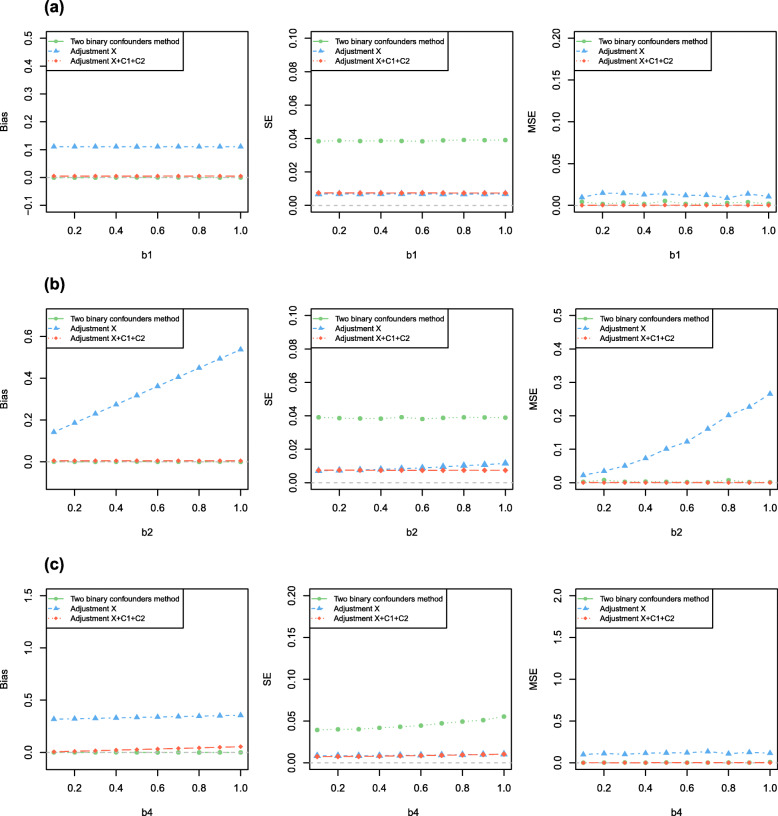
The Simulation A1 result. Results shows the estimated biases, *SE* and *MSE* from the 3 models for varied effects of **a***X* on *Y*, **b***C*_1_ on *Y*, **c***U* on *Y*

**Fig. 4:**
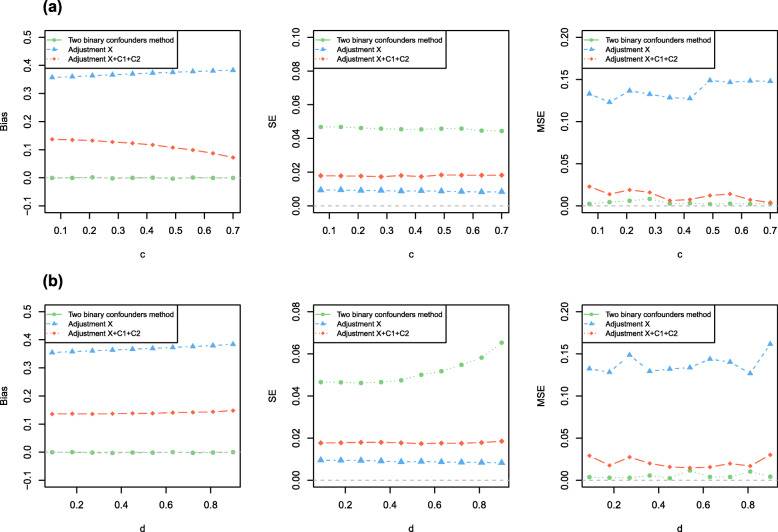
The Simulation A2 result. Results shows the estimated biases, *SE* and *MSE* from the 3 models for varied effects of **a** the correlation between *X* and *C*_1_, **b** the correlation between *C*_1_ and *C*_2_

We changed one parameter at a time while keeping all others at their basic values, when *N* = 2000 for Simulation A1-A2, *N* = 10,000 for Simulation B1-B2, *N* = 100,000 for Simulation C1-C2 and *N* = 100,000 for Simulation D1-D2. Results showed the estimated bias, standard error (*SE*) and the mean squared error (*MSE*) from the three models for varied effects of *C*_1_ on *X*, the interaction effects of *C*_1_ and *C*_2_ on *X*, *U* on *X*, *X* on *Y*, *C*_1_ on *Y*, *U* on *Y*, and the correlations among *C*_1_, *C*_2_ and *U*. The results showed the estimates of the model without any adjustment (model 1) and the model adjusting for both *C*_1_ and *C*_2_ (model 2) were biased. When we used the method we proposed (model 3) to analyze simulated data sets, the estimates were unbiased and had acceptable standard error. And our method had better *MSE* than other methods (i.e. model 1 and model 2). Figures 2 and 3 showed the results of Simulation A1, Fig. 4 showed the partial results of Simulation A2, the rest of results of Simulation A2 and other six different scenarios were showed in Figure S1, S2, S3, S4, S5, S6, S7, S8, S9, S10, S11, S12, S13 (Figure S1-S2 for Simulation A2, Figure S3-S4 for Simulation B1, Figure S5, S6, S7 for Simulation B2, Figure S8-S9 for Simulation C1, Figure S10 for Simulation C2, Figure S11-S12 for Simulation D1, Figure S13 for Simulation D2). Specifically, when varying across the effects of *C*_1_ on *Y*, the biases of unadjusted model (model 1) had a significant positive linear correlation with *β*_2_ and the ones of adjustment for *C*_1_ and *C*_2_ (model 2) remained stable. When varying across the effects of *U* on *Y*, the biases of both model 1 and model 2 linearly increased. Our method still had unbiased causal effect estimates and lower *MSE* than other two methods. Similarly, when varying across the effects of *C*_1_, *U* on *X*, respectively, the biases of model 1 and model 2 monotonically varied. Furthermore, the larger interaction effect of *C*_1_ and *C*_2_ on *X*, the causal effect estimation had higher precision for our proposed method (model 3). In order to make sure model 3 remains the smallest *MSE* among three models, we suggested the interaction effect of *C*_1_ and *C*_2_ on *X* moderately larger. Certainly, the bias did not change significantly from the basic scenario in any of the models when we changed the causal effect of *X* on *Y*. Moreover, when there existed an correlation between observed confounders *C*_1_ and *C*_2_, our method still got unbiased causal effect estimated. We still obtained the almost unbiased causal effect estimation if *P*(*C*_1_ = 1) = *P*(*C*_2_ = 1) = 0.5 when observed confounder *C*_1_ (or *C*_2_) and unobserved confounders *U* were correlated.

Compared to the situation of continuous variables, sample size *N* needs to be larger for discrete exposure and outcome variable. Under the linear probability model setting of *X*, *C*_1_, *C*_2_, *U* on *Y* (i.e. 0 ≤ *P*(*Y*| *X*, *C*_1_, *C*_2_, *U*) ≤ 1), the effects of *C*_1_ on *X*, *C*_2_ on *X*, the interaction effect of *C*_1_ and *C*_2_ on *X*, *U* on *X* should be relatively small. Furthermore, our method had better *MSE* than model 2, and was similar with model 1, the estimates remained unbiased. We use R program (version 3.6.1) to reproduce all simulations and analyses which are available on Github (https://github.com/LULIU1816/Two-binary-confounder).

### Data application results

In this section, we used the method proposed in this article to evaluate the potential causal effects of BMI on SBP, DBP, FBG, TG, TC, HDL and LDL using data from a follow-up survey in Jining, Shandong Province. Furthermore, we selected age and gender as near-IVs compared with the traditional regression approaches adjusting for age (discrete) and gender, adjusting for age (continuous) and gender and adjusting for age (discrete), gender and other health factors (including SBP, DBP, FBG, TC, TG, HDL and LDL). The cohort recruited 136,895 individuals aged 20- years between 2007 and 2015. In order to avoid reverse causation, we selected the data of BMI, age, gender in 2014 and SBP, DBP, FBG, TG, TC, HDL and LDL in 2015(*N* = 7013), respectively. Age and gender were divided into two discrete variables 0, 1. Age was discretized by median 40 as 0 and 1. Subject characteristics showed at Table 1. Proportion of female was 41.01%. Kolmogorov-Smirnov test is one of normality tests when the sample size over 5000. The median SBP was 120 (95% CI: 109–160) mmHg, the median DBP was 75 (95% CI: 66–101) mmHg. The median FBG was 5.20 (95% CI: 4.80–8.20) mmol/L, the median TC was 4.64 (95% CI: 4.01–6.76) mmol/L, the median TG was 1.04 (95% CI: 0.60–4.67) mmol/L, the median HDL was 1.29 (95% CI: 1.10–1.87) mmol/L, the median LDL was 2.76 (95% CI: 2.23–4.30) mmol/L. And, the median BMI was 24.68 (95% CI: 21.83–32.07) kg/m^2^.

**Table 1:** Subjects characteristics in Jining, Shandong Province

Variables	*N* = 7013	P^a^
Age (years, Median(95% CI))	40(31–70)	< 2.2×10^-16^
Female (n, %)	2876 (41.01%)	
SBP (mmHg, Median(95% CI))	120 (109–160)	< 2.2×10^-16^
DBP (mmHg, Median(95% CI))	75 (66–101)	< 2.2×10^-16^
FBG (mmol/L, Median(95% CI))	5.20 (4.80–8.20)	< 2.2×10^-16^
TC (mmol/L, Median(95% CI))	4.64 (4.01–6.76)	< 2.2×10^-16^
TG (mmol/L, Median(95% CI))	1.04 (0.60–4.67)	< 2.2×10^-16^
HDL (mmol/L, Median(95% CI))	1.29 (1.10–1.87)	< 2.2×10^-16^
LDL (mmol/L, Median(95% CI))	2.74 (2.23–4.30)	< 2.2×10^-16^
BMI (kg/m^2^, Median(95% CI))	24.68 (21.83–32.07)	6.96×10^-07^

^a^Kolmogorov-Smirnov test

Results of our proposed method and the traditional regression approach adjusting for age and gender are showed in Table 2. Firstly, we examine whether two independent or correlated available confounders satisfy the non-linear condition on the exposure. Results confirmed the interaction between age and gender on BMI (*P* = 2.82 × 10^−9^). Traditional regression approach implied the significant associations between SBP, DBP, FBG, TG, TC, HDL, LDL and BMI, respectively. BMI had obviously positive correlation with SBP, DBP, FBG, TG, TC and LDL while obviously negative correlation with HDL. However, our proposed method revealed the causal effect of BMI on indicators including SBP, DBP, TG, TC, LDL. In addition, the causal effects of BMI on HDL and FBG were not significant. SBP increased 1.60 (95% CI: 0.99–2.93) mmol/L with per 1- kg/m^2^ higher BMI and DBP increased 0.37 (95% CI: 0.03–0.76) mmol/L with per 1- kg/m^2^ higher BMI. Moreover, 1- kg/m^2^ increase in BMI had potential causality with a 1.61-SD increase in TC (*β*, 1.61; 95% CI: 0.96–2.97), a 1.66-SD increase in TG (*β*, 1.66; 95% CI: 0.91–55.30) and a 2.01-SD increase in LDL (*β*, 2.01; 95% CI: 1.09–4.31). However, BMI had no potential causality with HDL (*β*, − 0.20; 95% CI: − 1.71-1.44). The effect of FBG per 1- kg/m^2^ higher BMI was 0.56 (95% CI: − 0.24-2.18). To conclude, the potential causal effects of BMI on SBP, DBP, FBG, TG, TC, HDL and LDL were almost consistent with previous studies. Furthermore, the results showed the robustness of the novel method.

**Table 2:** The causal effect of BMI on SBP, DBP, FBG, TG, TC, HDL and LDL

	double confounding variables model			regression adjusting for age (discrete) and gender		
	β	SE	95%CI	β	SE	*P*-value
BMI → SBP	1.60	0.62	0.99–2.93	0.25	0.01	3.83×10^-74^
BMI → DBP	0.37	0.18	0.03–0.76	0.22	0.01	3.90×10^-51^
BMI → FBG	0.56	0.86	−0.24-2.18	0.11	0.02	1.98×10^-09^
BMI → TC	1.61	0.58	0.96–2.97	0.11	0.02	5.67×10^-13^
BMI → TG	1.66	1.36	0.91–55.30	0.27	0.02	2.19×10^-51^
BMI → HDL	−0.20	0.92	−1.71-1.44	−0.20	0.02	4.12×10^-19^
BMI → LDL	2.01	0.84	1.09–4.31	0.12	0.02	2.21×10^-11^
	regression adjusting for age (discrete), gender and other health factors			regression adjusting for age (continuous) and gender		
	β	SE	*P*-value	β	SE	*P*-value
BMI → SBP	0.15	0.02	< 2×10^-16^	0.24	0.01	4.19×10^-70^
BMI → DBP	0.10	0.02	1.67×10^-07^	0.22	0.01	1.63×10^-50^
BMI → FBG	0.07	0.02	1.58×10^-03^	0.10	0.02	1.66×10^-09^
BMI → TC	−0.02	0.02	0.23	0.11	0.02	1.87×10^-12^
BMI → TG	0.09	0.02	1.57×10^-05^	0.27	0.02	6.24×10^-51^
BMI → HDL	−0.11	0.03	8.71×10^-06^	−0.20	0.02	7.45×10^-19^
BMI → LDL	0.04	0.02	0.02	0.12	0.02	5.12×10^-11^

## Discussion

In this paper, we present a simple and intuitive method to identify and correct for confounding bias in observation studies using a confounder–exposure nonlinear condition. In cases where the independence assumption between observed confounder and unobserved confounder is violated, a sensible approach shows the almost unbiased causal effect estimation if *P*(*C*_1_ = 1) = *P*(*C*_2_ = 1) = 0.5. In this sense, our proposed method can be viewed very much as a tool for identifying causal effect in epidemiology.

To identify and estimate causal effect with unobserved confounders, different approaches require different untestable assumptions, such as DID needs an untestable common trend assumption. Fortunately, the approach proposed in this paper requires the nonlinearity assumption of at least two observed confounders (*C*_1_, *C*_2_, ⋯, *C*_*l*_) and treatment variable *X*, as well as the conditional expectation of unobserved confounders given observed confounders *U* is 0. Furthermore, the observed data contain (*C*_1_, *C*_2_, ⋯, *C*_*l*_) and *X* which can be utilized to test our nonlinearity assumption. This is exclusive to our method compared with other methods.

For binary variable, the parameter estimates from the LPM can be directly interpreted as the effect of the exposure on the prevalence rate of the outcome which is consistent with the ACE. Conversely, logistic regression or log-linear regression are not applicable in causal inference, since they do not provide a direct interpretation of bivariate associations. However, error terms of LPM with ordinary least square estimation are heteroskedastic and predicted probability can be above 1 or below 0. Therefore, we adopt GMM to deal with these problems. Additionally, our proposed method has no assumption on the independence among observed confounders. This can be widely used in epidemiology to obtain the causal inference of the exposure on the outcome.

Another advantage is the accessibility of two observed confounders satisfying nonlinear condition. Further, we find out that using GMM still fairly reliable in our proposed method if the number of observed confounders is over two.

A number of factors must be considered before implementing the method. First, the independence assumption between observed and unobserved confounders is essential for causal estimate correction. One strategy to overcome this limitation is to select other observed confounders with the probabilities of binary values 0.5. Second, interaction between observed confounders and unobserved confounders on exposure is sufficiently strong. Finally, our method cannot identify the reverse causation. Requiring a priori knowledge or using the idea of Cross-lagged Panel Analysis may avoid this problem. Identifying causal effects across studies of differing design can therefore prove valuable in further research, whilst agreement with the result of Mendelian Randomization and randomization experiment strengthens confidence in the resulting findings and subsequent inference.

## Conclusions

In conclusions, we propose a novel method to control unobserved confounding through double or more binary confounders satisfying a non-linear condition on the exposure which is easy to access. In particular, our method can handle general cases regardless of a continuous or categorical exposure and outcome. Various simulations show better estimation performance by our approach and suggest that our method will be more widely used in observational studies to explore causal association.

## Supplementary information

**Additional file 1 **: **Appendix A.** Proof of Theorem 1.

**Additional file 2 **: **Appendix B.** Proof for the equivalence of different choices of *f*(·) in Eq. (4) for the estimation when the identifiability condition in Theorem 1 holds and Proof for matrix *Q*^eff^ equals *E*[*f*(*C*_1_, *C*_2_)*f*(*C*_1_, *C*_2_)^*T*^] in Data application results section.

**Additional file 3 **: **Appendix C.** Simulation parameter settings.

**Additional file 4 **: **Figure S1.** The Simulation A2 result. Results shows the estimated biases, *SE* and *MSE* from the 3 models for varied effects of (a) *C*_1_ on *X*, (b) the interaction effect of *C*_1_ and *C*_2_ on *X*, (c) *U* on *X*.

**Additional file 5 **: **Figure S2.** The Simulation A2 result. Results shows the estimated biases, *SE* and *MSE* from the 3 models for varied effects of (a) *X* on *Y*, (b) *C*_1_ on *Y*, (c) *U* on *Y.*

**Additional file 6 **: **Figure S3.** The Simulation B1 result. Results shows the estimated biases, *SE* and *MSE* from the 3 models for varied effects of (a) *C*_1_ on *X*, (b) the interaction effect of *C*_1_ and *C*_2_ on *X*, (c) *U* on *X*.

**Additional file 7 **: **Figure S4.** The Simulation B1 result. Results shows the estimated biases, *SE* and *MSE* from the 3 models for varied effects of (a) *X* on *Y*, (b) *C*_1_ on *Y*, (c) *U* on *Y.*

**Additional file 8 **: **Figure S5** The Simulation B2 result. Results shows the estimated biases, *SE* and *MSE* from the 3 models for varied effects (a) of *C*_1_ on *X*, (b) the interaction effect of *C*_1_ and *C*_2_ on *X*, (c) *U* on *X*.

**Additional file 9 **: **Figure S6.** The Simulation B2 result. Results shows the estimated biases, *SE* and *MSE* from the 3 models for varied effects of (a) *X* on *Y*, (b) *C*_1_ on *Y*, (c) *U* on *Y.*

**Additional file 10 **: **Figure S7.** The Simulation B2 result. Results shows the estimated biases, *SE* and *MSE* from the 3 models for varied effects of (a) the correlation between *X* and *C*_1_, (b) the correlation between *C*_1_ and *C*_2_.

**Additional file 11 **: **Figure S8.** The Simulation C1 result. Results shows the estimated biases, *SE* and *MSE* from the 3 models for varied effects (a) of *C*_1_ on *X*, (b) the interaction effect of *C*_1_ and *C*_2_ on *X*, (c) *U* on *X*.

**Additional file 12 **: **Figure S9.** The Simulation C1 result. Results shows the estimated biases, *SE* and *MSE* from the 3 models for varied effects of (a) *X* on *Y*, (b) *C*_1_ on *Y*, (c) *U* on *Y.*

**Additional file 13 **: **Figure S10.** The Simulation C2 result. Results shows the estimated biases, *SE* and *MSE* from the 3 models for varied effects of (a) the correlation between *X* and *C*_1_, (b) the correlation between *C*_1_ and *C*_2_.

**Additional file 14 **: **Figure S11.** The Simulation D1 result. Results shows the estimated biases, *SE* and *MSE* from the 3 models for varied effects of (a) *C*_1_ on *X*, (b) the interaction effect of *C*_1_ and *C*_2_ on *X*, (c) *U* on *X*.

**Additional file 15 **: **Figure S12.** The Simulation D1 result. Results shows the estimated biases, *SE* and *MSE* from the 3 models for varied effects of (a) *X* on *Y*, (b) *C*_1_ on *Y*, (c) *U* on *Y.*

**Additional file 16 **: **Figure S13.** The Simulation D2 result. Results shows the estimated biases, *SE* and *MSE* from the 3 models for varied effects of (a) the correlation between *X* and *C*_1_*,* (b) the correlation between *C*_1_ and *C*_2_.

## Data Availability

The datasets used and/or analyzed during the present study are available from the corresponding author on reasonable request.

## Abbreviations

IPW: Inverse probability weighing
BMI: Body mass index
SBP: Systolic blood pressure
DBP: Diastolic blood pressure
FBG: Fasting blood glucose
TG: Triglyceride
TC: Total cholesterol
HDL: High density lipoprotein
LDL: Low density lipoprotein
IVs: Instrument variables
IVA: Instrumental variable analysis
DID: Difference-in-differences
RDD: Regression discontinuity design
ACE: Average causal effect
GMM: Generalized moment estimate model
SE: Standard error
MSE: Mean squared error
LPM: Linear probability model
