# Supplementary material for: A novel method for controlling unobserved confounding using double confounders
Source: BMC Med Res Methodol. 2020 Jul 22;20:195. doi: 10.1186/s12874-020-01049-0 (PMC7374896; doi:10.1186/s12874-020-01049-0)
Supplement: Supplementary file 1 — Additional file 1 : Appendix A. Proof of Theorem 1. [file 12874_2020_1049_MOESM1_ESM.docx]

**Appendix A**

**Proof of Theorem 1:** We separately show the necessity and sufficiency for the identifiability of parameters in model (1).

For necessity, suppose that the non-linearity condition does not hold, that is, $|\rho(E(X|C_{1},C_{2}),C_{1})|=1$, $|\rho(E(X|C_{1},C_{2}),C_{2})|=1$, or$|\rho(C_{1},C_{2})|=1$. Think about one of them, $|\rho(E(X|C_{1},C_{2}),C_{1})|=1$, others are similar to obtain the same result. This implies that there exist some$\tau_{0}$and$\tau_{1}$satisfying $E(X|C_{1},C_{2})=\tau_{0}+\tau_{1}C_{1}$ almost everywhere. Then from the model (2) we have

$$\begin{matrix} E(Y|C_{1},C_{2})=\beta_{0}+\beta_{1}E(X|C_{1},C_{2})+\beta_{2}C_{1}+\beta_{3}C_{2} \\ \text{ } =(\beta_{0}+\tau_{0}\beta_{1})+(\tau_{1}\beta_{1}+\beta_{2})C_{1}+\beta_{3}C_{2} \end{matrix}$$

The above equation implies that *Y* is marginally linear with respect to $C_{1}$ and $C_{2}$. For this linear model, only$\beta_{0}+\tau_{0}\beta_{1}$,$\tau_{1}\beta_{1}+\beta_{2}$,$\beta_{3}$are identifiable as a whole, while parameters$\tau_{0},\tau_{1},\beta_{0},\beta_{1},\beta_{2},\beta_{3}$ cannot be distinguished each other.

For sufficiency, if *X* is not marginally linearly related with respect to $C_{1}$ and $C_{2}$, and the conditional expectation of *U* given $C_{1}$ and $C_{2}$ is a constant.

Let $\beta_{0}^{T}=\beta_{0}+\beta_{4}E\left(U|C_{1},C_{2}\right)$, $\beta=\left(\beta_{0},\beta_{1},\beta_{2},\beta_{3}\right)^{T}$, $Q=\left(1,E\left(X|C_{1},C_{2}\right),C_{1},C_{2}\right)$, $R=\left(E\left(Y|C_{1},C_{2}\right)\right)$, we can obtain

$Q\times\beta=R$ (A.1)

Then we can find four levels for$C_{1}$and$C_{2}$,$C_{1}=0,\text{ }C_{2}=0;\text{ }C_{1}=0,\text{ }C_{2}=1;\text{ }C_{1}=1,\text{ }C_{2}=0;\text{ }C_{1}=1,\text{ }C_{2}=1$, which satisfy$\left|Q\right|=\left\{ \left[E\left(X|C_{1}=0\text{,}C_{2}=1\right)-E\left(X|C_{1}=0\text{,}C_{2}=0\right)\right]-\left[E\left(X|C_{1}=1\text{,}C_{2}=1\right)-E\left(X|C_{1}=1\text{,}C_{2}=0\right)\right]\right\}\neq0$, that is the matrix $Q$has full rank.

Thus parameters $\beta_{0},\beta_{1},\beta_{2},\beta_{3}$ can be identified. Among them, the parameter $\beta_{1}$ can be identified from

$\beta_{1}\text{=}\frac{\left[E\left(Y|C_{1}=1,C_{2}=1\right)-E\left(Y|C_{1}=1,C_{2}=0\right)\right]-\left[E\left(Y|C_{1}=0\text{,}C_{2}=1\right)-E\left(Y|C_{1}=0\text{,}C_{2}=0\right)\right]}{\left[E\left(X|C_{1}=0\text{,}C_{2}=1\right)-E\left(X|C_{1}=0\text{,}C_{2}=0\right)\right]-\left[E\left(X|C_{1}=1\text{,}C_{2}=1\right)-E\left(X|C_{1}=1\text{,}C_{2}=0\right)\right]}$ (A.2)□
