# Supplementary material for: A novel method for controlling unobserved confounding using double confounders
Source: BMC Med Res Methodol. 2020 Jul 22;20:195. doi: 10.1186/s12874-020-01049-0 (PMC7374896; doi:10.1186/s12874-020-01049-0)
Supplement: Supplementary file 2 — Additional file 2 : Appendix B. Proof for the equivalence of different choices of f(·) in Eq. (4) for the estimation when the identifiability condition in Theorem 1 holds and Proof for matrix Qeff equals E[f(C1, C2)f(C1, C2)T] in Data application results section. [file 12874_2020_1049_MOESM2_ESM.docx]

**Appendix B**

Proof for the equivalence of different choices of in equation (4) for the estimation when the identifiability condition in Theorem 1 holds

We want to show that an arbitrary vector function that identifies $\beta$ via equation (4) leads to the same estimator as that based on the function $f^{\text{*}}\left(C_{1},C_{2}\right)$. For an arbitrary vector function ($K>2$), we can denote it as$f\left(C_{1},C_{2}\right)=\left[\begin{matrix} f_{1}\left(C_{1}=0,C_{2}=0\right) f_{2}\left(C_{1}=0,C_{2}=1\right) f_{3}\left(C_{1}=1,C_{2}=0\right) f_{4}\left(C_{1}=1,C_{2}=1\right) \\ \vdots\vdots\vdots\vdots\\ f_{K}\left(C_{1}=0,C_{2}=0\right) f_{K}\left(C_{1}=0,C_{2}=1\right) f_{K}\left(C_{1}=1,C_{2}=0\right) f_{K}\left(C_{1}=1,C_{2}=1\right) \end{matrix}\right]\left[\begin{matrix} \delta\left(C_{1}\text{=}0,C_{2}\text{=}0\right) \\ \delta\left(C_{1}\text{=}0,C_{2}\text{=}1\right) \\ \delta\left(C_{1}\text{=}1,C_{2}\text{=}0\right) \\ \delta\left(C_{1}\text{=}1,C_{2}\text{=1}\right) \end{matrix}\right]$ (A.3)

Let $P$ denote the $K\times3$ matrix on the right hand side. Equation (A.1) can be rewritten as $Q\times\beta=R$, where $Q=E\left(f\left(C_{1},C_{2}\right),Xf\left(C_{1},C_{2}\right),C_{1}f\left(C_{1},C_{2}\right),C_{2}f\left(C_{1},C_{2}\right)\right)$ and $R=E\left(Yf\left(C_{1},C_{2}\right)\right)$. Then the estimation equation for $\beta$ is $\widehat{\beta}=\widehat{Q}^{-1}\widehat{R}$. From (A.3), we have

$\begin{matrix} \widehat{Q}\text{=}\widehat{E}\left(f\left(C_{1},C_{2}\right),Xf\left(C_{1},C_{2}\right),C_{1}f\left(C_{1},C_{2}\right),C_{2}f\left(C_{1},C_{2}\right)\right) \\ =\widehat{E}\left(Pf^{*}\left(C_{1},C_{2}\right),XPf^{*}\left(C_{1},C_{2}\right),C_{1}Pf^{*}\left(C_{1},C_{2}\right),C_{2}Pf^{*}\left(C_{1},C_{2}\right)\right) \\ =P\widehat{E}\left(f^{*}\left(C_{1},C_{2}\right),Xf^{*}\left(C_{1},C_{2}\right),C_{1}f^{*}\left(C_{1},C_{2}\right),C_{2}f^{*}\left(C_{1},C_{2}\right)\right) \\ =P\widehat{Q^{*}} \end{matrix}$

where  denotes the sample mean of the corresponding variable. Similarly, we have $\widehat{R}=P\widehat{R^{*}}$*.*

Then by the function , the estimation equation for $\beta$ is equivalent to

$\widehat{Q}\widehat{\beta}-\widehat{R}\text{=}P\left(\widehat{Q^{*}}\widehat{\beta}-\widehat{R^{*}}\right)=0$.

Since $\widehat{\beta^{*}}=\widehat{Q^{*}}^{-1}\widehat{R^{*}}$satisfies the equation $Q^{*}\beta-R^{*}=0$, we have that $\widehat{\beta^{*}}$also satisfies $\widehat{Q}\widehat{\beta^{*}}-\widehat{R}=0$. Thus we proved $\widehat{\beta}\text{=}\widehat{\beta^{*}}$ when *P* has full rank, which means that the above equation of $\widehat{\beta}$ has a unique solution.

Proof for **matrix $Q^{\text{eff}}$ equals $E\left[f\left(C_{1},C_{2}\right)f\left(C_{1},C_{2}\right)^{T}\right]$ in section 3.2**

We show that $Q^{\text{eff}}=E\left[f\left(C_{1},C_{2}\right)f\left(C_{1},C_{2}\right)^{T}\right]$**.** It is obvious that

$$\begin{matrix} E\left[f\left(C_{1},C_{2}\right)f\left(C_{1},C_{2}\right)^{T}\right]\text{=}E\left[f^{\text{eff}}\left(C_{1},C_{2}\right)f^{\text{eff}}\left(C_{1},C_{2}\right)^{T}\right] \\ \text{ =}\left[\begin{matrix} \text{ }1\text{ }E\left(X\right)\text{ }E\left(C_{1}\right)\text{ }E\left(C_{2}\right) \\ E\left(X\right)\text{ }E\left[E\left(X|C_{1},C_{2}\right)^{2}\right]\text{ }E\left(C_{1}X\right)\text{ }E\left(C_{2}X\right) \\ E\left(C_{1}\right)\text{ }E\left(C_{1}X\right)\text{ }E\left(C_{1}^{2}\right)\text{ }E\left(C_{1}C_{2}\right) \\ E\left(C_{2}\right)\text{ }E\left(C_{2}X\right)\text{ }E\left(C_{1}C_{2}\right)\text{ }E\left(C_{2}^{2}\right) \end{matrix}\right]\text{=}Q^{\text{eff}} \end{matrix}$$
