# Supplementary material for: A novel method for controlling unobserved confounding using double confounders
Source: BMC Med Res Methodol. 2020 Jul 22;20:195. doi: 10.1186/s12874-020-01049-0 (PMC7374896; doi:10.1186/s12874-020-01049-0)
Supplement: Supplementary file 3 — Additional file 3 : Appendix C. Simulation parameter settings. [file 12874_2020_1049_MOESM3_ESM.docx]

**Appendix C**

Compared to Simulation A1-A2, Simulation B1-B2 are different with two steps:

(1)Draw $U$from a uniform distribution $U\left(0,1\right)$ for Simulation B1-B2; Draw *U* from the third column of multivariate normal distribution, then obtained empirical cumulative distribution function of $U$as a distribution of $U$.

(2)Draw$X$from a normal distribution with a linear probability model,

$$P\left(X|C_{1},C_{2}\right)\text{=}\alpha_{0}+\alpha_{1}C_{1}+\alpha_{2}C_{2}+\alpha_{3}C_{1}C_{2}+\alpha_{4}U$$

And the parameters are set as follows: $\left(p_{1},p_{2},\alpha_{1},\alpha_{2},\alpha_{3},\alpha_{4},\beta_{1},\beta_{2},\beta_{3},\beta_{4},c,d\right)=\left(0.5,0.5,0.2,0.1,0.3\text{，}0.1,1,0.5,0.3,0.1,0.1,0.2\right)$

For Simulation C1-C2 and D1-D2, we draw $U$ and $\varepsilon_{X}$ from uniform distribution $U\left(-1,1\right)$ and $U\left(-0.1,0.1\right)$to limit$X$to a certain range [0,1], respectively. In addition, the discrete variables are generate with a linear probability model instead of linear model.

And the parameters are set as follows: $\left(p_{1},p_{2},\alpha_{1},\alpha_{2},\alpha_{3},\alpha_{4},\beta_{1},\beta_{2},\beta_{3},\beta_{4},c,d\right)=\left(0.5,0.5,0.1,0.1,0.2\text{，}0.1,0.5,0.1,0.1,0.1,0.5,0.5\right)$
