# Supplementary material for: A novel method for controlling unobserved confounding using double confounders
Source: BMC Med Res Methodol. 2020 Jul 22;20:195. doi: 10.1186/s12874-020-01049-0 (PMC7374896; doi:10.1186/s12874-020-01049-0)
Supplement: Supplementary file 7 — Additional file 7 : Figure S4. The Simulation B1 result. Results shows the estimated biases, SE and MSE from the 3 models for varied effects of (a) X on Y, (b) C1 on Y, (c) U on Y. [file 12874_2020_1049_MOESM7_ESM.pdf]

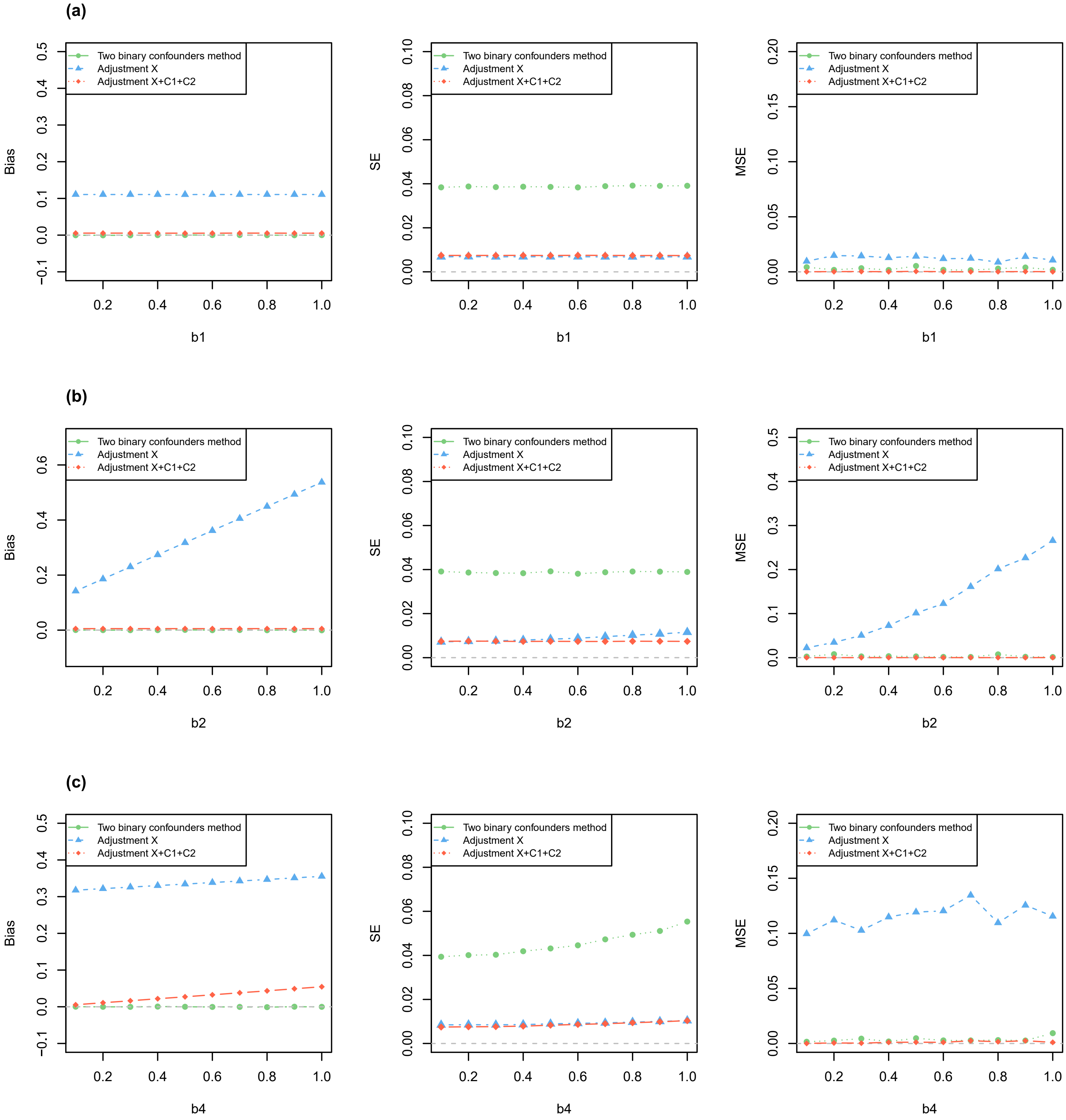

Figure S4 The Simulation B1 result. Results shows the estimated biases, *SE* and *MSE* from the 3 models for varied effects of (a) X on Y, (b) C_1 on Y, (c) U on Y.
