# Supplementary material for: A novel method for controlling unobserved confounding using double confounders
Source: BMC Med Res Methodol. 2020 Jul 22;20:195. doi: 10.1186/s12874-020-01049-0 (PMC7374896; doi:10.1186/s12874-020-01049-0)
Supplement: Supplementary file 10 — Additional file 10 : Figure S7. The Simulation B2 result. Results shows the estimated biases, SE and MSE from the 3 models for varied effects of (a) the correlation between X and C1, (b) the correlation between C1 and C2. [file 12874_2020_1049_MOESM10_ESM.pdf]

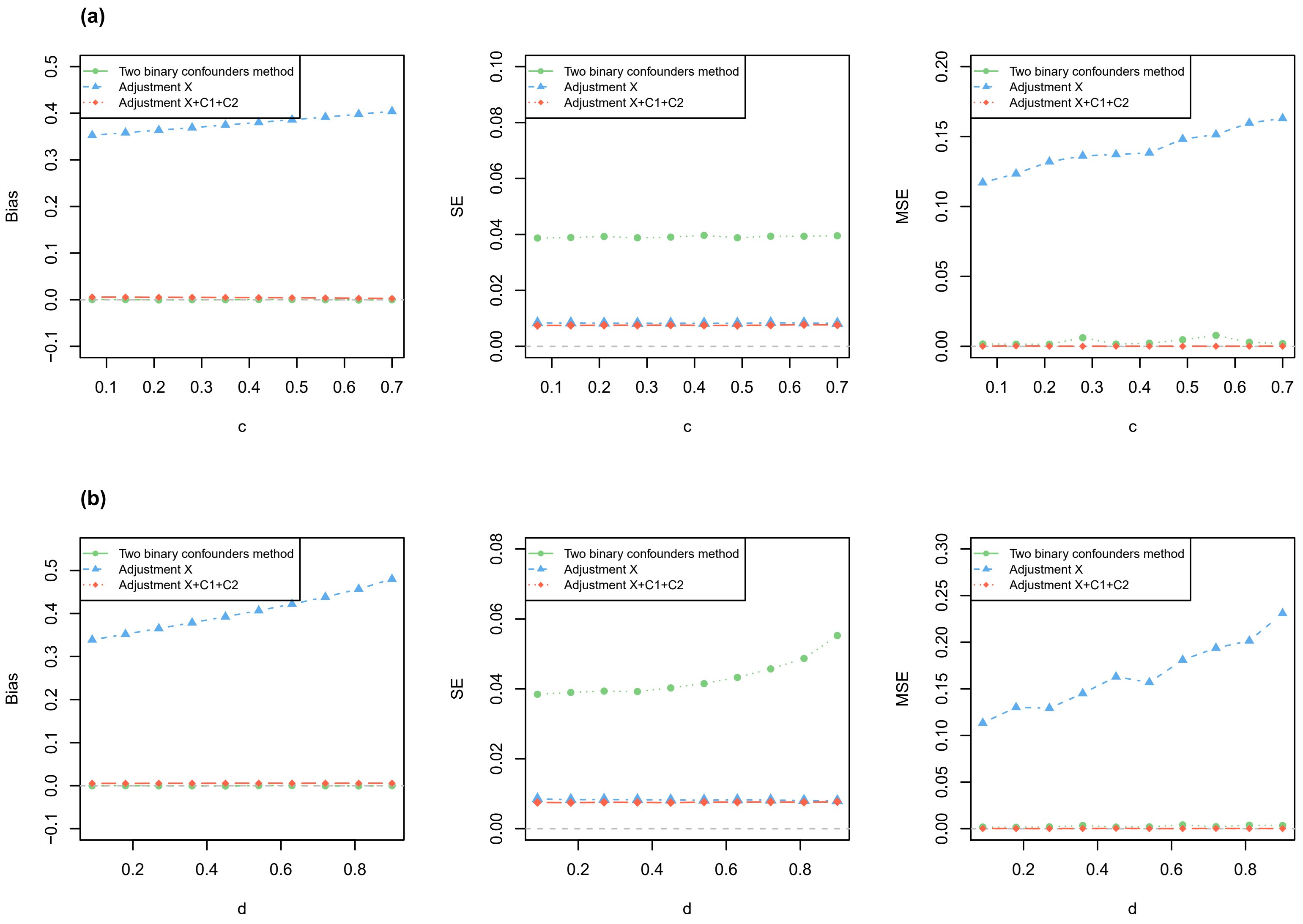

Figure S7 The Simulation B2 result. Results shows the estimated biases, *SE* and *MSE* from the 3 models for varied effects of (a) the correlation between C_1 and X, (b) the correlation between C_1 and C_2.
