# Supplementary material for: A novel method for controlling unobserved confounding using double confounders
Source: BMC Med Res Methodol. 2020 Jul 22;20:195. doi: 10.1186/s12874-020-01049-0 (PMC7374896; doi:10.1186/s12874-020-01049-0)
Supplement: Supplementary file 11 — Additional file 11 : Figure S8. The Simulation C1 result. Results shows the estimated biases, SE and MSE from the 3 models for varied effects (a) of C1 on X, (b) the interaction effect of C1 and C2 on X, (c) U on X. [file 12874_2020_1049_MOESM11_ESM.pdf]

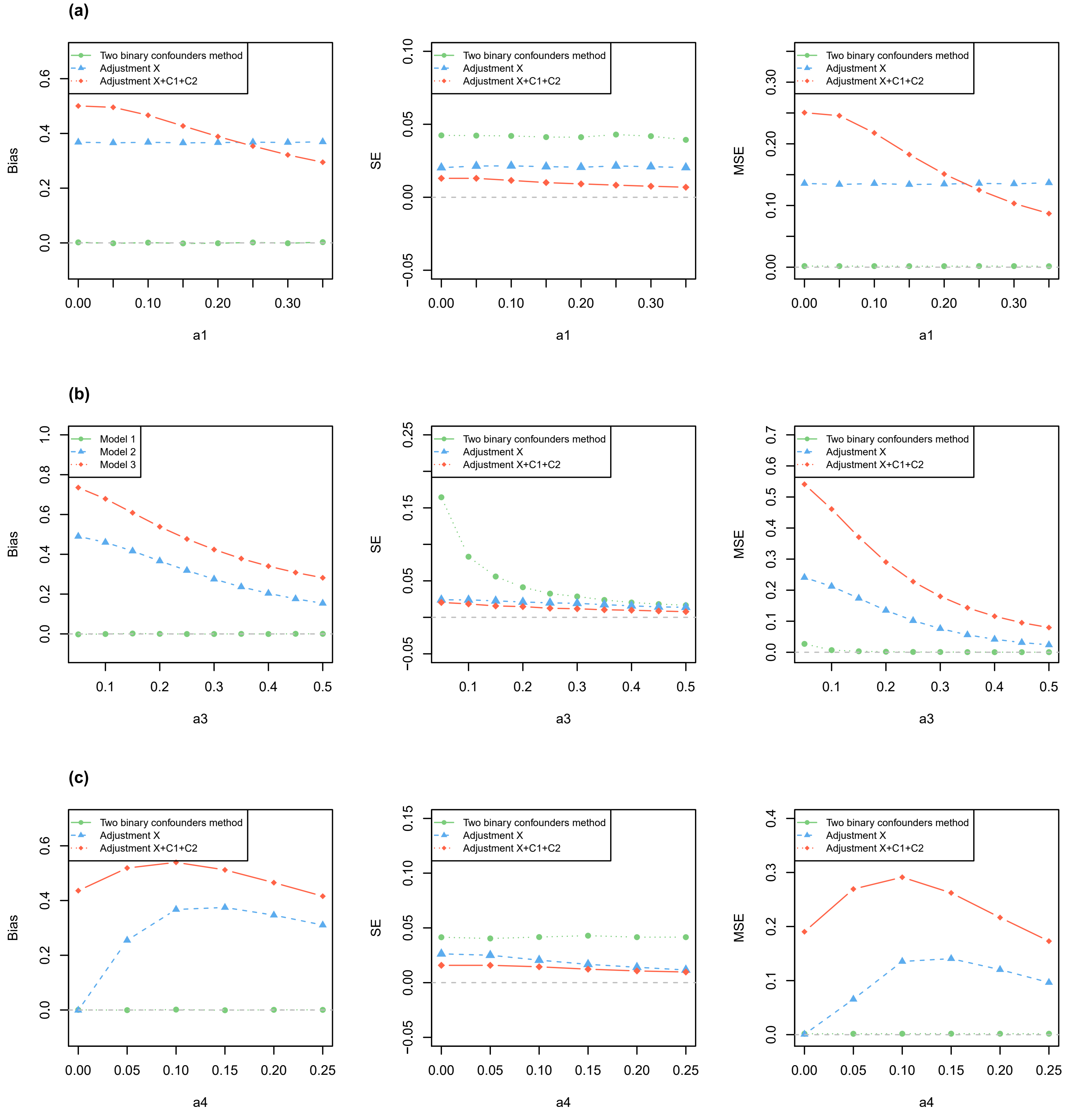

Figure S8 The Simulation C1 result. Results shows the estimated biases, *SE* and *MSE* from the 3 models for varied effects (a) of C_1 on X, (b) the interaction effect of C_1 and C_2 on X, (c) U on X.
